# Electronic Voting Protocol Using Identity-Based Cryptography

**DOI:** 10.1155/2015/741031

**Published:** 2015-05-24

**Authors:** Gina Gallegos-Garcia, Horacio Tapia-Recillas

**Affiliations:** ^1^Sección de Estudios de Posgrado e Investigación, Escuela Superior de Ingeniería Mecánica y Eléctrica, Instituto Politécnico Nacional, Avenida Santa Ana 1000, San Francisco, Culhuacan, Coyoacan, 04430 México City, DF, Mexico; ^2^Departamento de Matematicas, Universidad Autónoma Metropolitana Iztapalapa, San Rafael Atlixco 186, Vicentina, Iztapalapa, 09340 México City, DF, Mexico

## Abstract

Electronic voting protocols proposed to date meet their properties based on Public Key Cryptography (PKC), which offers high flexibility through key agreement protocols and authentication mechanisms. However, when PKC is used, it is necessary to implement Certification Authority (CA) to provide certificates which bind public keys to entities and enable verification of such public key bindings. Consequently, the components of the protocol increase notably. An alternative is to use Identity-Based Encryption (IBE). With this kind of cryptography, it is possible to have all the benefits offered by PKC, without neither the need of certificates nor all the core components of a Public Key Infrastructure (PKI). Considering the aforementioned, in this paper we propose an electronic voting protocol, which meets the privacy and robustness properties by using bilinear maps.

## 1. Introduction

Since 1964, considerable efforts have been made to improve the efficiency of election processes that has brought, as a consequence, a wide range of proposals on such topic.

Electronic voting has been mentioned in different media as the use of computers or computerized voting equipment to cast ballots in an election, which nowadays are a reasonable alternative to conventional elections and other opinion expressing processes [1–5]. Roughly speaking an electronic voting protocol, used to develop an electronic voting process, involves three main entities: voters, registration authorities, and counting authorities who interact with each other during four main phases: registration, authentication, voting, and counting [6, 7], from which authentication is out of our scope.

In order to use an electronic voting protocol inside an electronic voting process, it should satisfy several properties [8]. However, proposed protocol meets privacy and robustness properties by using bilinear maps.

- Privacy: a vote must be kept secret from any coalition of authorities.
- Robustness: the protocol can be developed even if there are entities who do not give correct information. In other words, this property is against dishonest users.

In this paper a voting protocol based on bilinear maps [9, 10] satisfying privacy, uncoercibility, and robustness is proposed. The paper is organized as follows: in Section 2 some intractable problems on finite groups are recalled. The security of the proposed protocol is based on these intractable problems. In Section 3 the proposed protocol is presented. An analysis of privacy and robustness properties is given in Section 4. Obtained results are showed in Section 5.  Section 6 presents concluding remarks and final references are listed.

## 2. Preliminaries

Let (*G*_1_, +) be a cyclic group of order *m* written additively. With such a group *G*_1_, the following hard cryptographic problems are defined:

- Discrete Logarithm Problem (DLP): given *P*, *P*′ ∈ *G*_1_, find an integer *n* such that *P* = *nP*′ whenever such integer exists.
- Computational Diffie-Hellman Problem (CDHP): given a triple *P*, *aP*, *bP* ∈ *G*_1_ for *a*, *b* ∈ *ℤ*_*m*_, find the element (*ab*)*P*.
- Decision Diffie-Hellman Problem (DDHP): given a quadruple *P*, *aP*, *bP*, *cP* ∈ *G*_1_ for *a*, *b*, *c* ∈ *ℤ*_*m*_, decide whether *c* ≡ *ab*(mod  *m*) or not.

We assume throughout the paper that DLP and CDHP are intractable, which means that there does not exist a Polynomial Time Algorithm to solve them with nonnegligible probability. When the DDHP is easy but the CDHP is hard on the group *G*_1_, *G*_1_ is called a Gap Diffie-Hellman (GDH) group. Such a group can be found on supersingular elliptic curves or hyperelliptic curves over finite fields [11, 12]. The proposed electronic voting protocol can be built on any GDH group.

## 3. The Proposed Electronic Voting Protocol

The protocol is divided into three phases: setup, voting, and counting. In the setup stage the key pairs to be used during the voting and counting phases are generated. The generation of these key pairs involves the participation of *n* entities *E*_*i*_, where 1 ≤ *i* ≤ *n* [12–14]. Each entity broadcasts and receives specific information by using Shamir's secret-sharing scheme in order to generate its public and private shares [15]. In the voting phase voters encrypt votes and ask a blind signature [13, 14]. In the counting phase, a Combining Entity reconstructs the signatures of the votes and verifies and decrypts them [13, 14, 16, 17].

The Combining Entity, who does not have any private key, decrypts the votes by combining decryption shares, which are generated by each entity *E*_*i*_, after which the votes are counted and the tally is published.

The three phases are detailed as follows.

### 3.1. Setup Phase

1. Let (*G*_1_, +) and (*G*_2_, *∗*) be cyclic groups of the same order *q* which is assumed to be a prime number, with *G*_1_ = 〈*P*_1_〉, and let $\hat{e}:G_{1}\times G_{1}\to G_{2}$ be a nondegenerated bilinear mapping. Let *H*_1_ : {0,1}^*∗*^ → *G*_1_ and *H*_2_ : *G*_2_ → {0,1}^*n*^ be two hash functions. This information is known to all entities *E*_*i*_, *i* = 1,2,…, *n*, where *n* ≤ *q* − 1. Furthermore, each entity *E*_*i*_ chooses a binary string, an element of {0,1}^*k*^, corresponding to information identifying this entity, for example, an e-mail address, an IP address, and telephone number. The entity *E*_*i*_ sends information to each *E*_*j*_ to generate the public encryption key *P*_pub_ and its respective private decryption key *d* as follows:
  - Entity *E*_*i*_ randomly selects *a*_*i*0_ ∈ *ℤ*_*q*_^*∗*^, keeps it in secret, and broadcasts *a*_*i*0_ *P*_1_.
  - Entity *E*_*i*_ randomly picks up a polynomial *f*_*i*_(*x*) = *a*_*i*0_ + *a*_*i*1_ *x* + ⋯+*a*_*it*−1_ *x* ^*t*−1^ ∈ *ℤ*_*q*_[*x*] of degree ≤*t* − 1 such that *f*_*i*_(0) = *a*_*i*0_. The integer *t* is taken sufficiently large.
  - *E*_*i*_ computes and broadcasts *a*_*ij*_ *P*_1_ for *j* = 1,2,…, *t* − 1 and sends *f*_*i*_(*j*) to each *E*_*j*_ for *j* = 1,2,…, *n*, where *j* ≠ *i*.
2. After *E*_*i*_ receives *f*_*j*_(*i*) from entity *E*_*j*_, *j* = 1,2,…, *n*,  *j* ≠ *i*, it does the following:
  - *E*_*i*_ verifies *f*_*j*_(*i*)*P*_1_ by checking that *f*_*j*_(*i*)*P*_1_ = (∑_*k*=0_^*t*−1^ *i* ^*k*^ *a*_*jk*_)*P*_1_, for each *j* = 1,2,…, *n*,  *j* ≠ *i*. If the check fails, *E*_*i*_ broadcasts a complaint against *E*_*j*_.
  - It computes its private share *d*_*i*_ = ∑_*k*=1_^*n*^ *f*_*k*_(*i*) and keeps it in secret. This *d*_*i*_ may be considered as an element of *ℤ*_*q*_.
3. Each *E*_*i*_ calculates its public share *P*_pub_*i*__ = *d*_*i*_ *P*_1_ ∈ *G*_1_ and computes the public encryption key *P*_pub_ = ∑_*i*=1_^*n*^ *a*_*i*0_ *P*_1_ ∈ *G*_1_.
4. With the above calculations, the public key is *P*_pub_ = *dP*_1_ and its respective private key, that is distributed to every entity *E*_*i*_, is *d* = ∑_*i*=1_^*n*^ *a*_*i*0_.
5. Let ID be the binary sequence identifying the receiver, also called Combining Entity, and let *P*_pub_ID__ = *H*_1_(ID) ∈ *G*_1_; all entities *E*_*i*_ compute their private encryption private share *d*_ID_*i*__ = *a*_*i*0_ *P*_pub_ID__.
6. In order to generate the signature and verification key pair, each entity *E*_*i*_ sends the following information to each *E*_*j*_. This is done by using the same (additive) group *G*_1_ = 〈*P*_1_〉 as follows:
  - Entity *E*_*i*_ randomly selects *b*_*i*0_ ∈ *ℤ*_*q*_^*∗*^, keeps it in secret, and broadcasts *b*_*i*0_ *P*_1_.
  - It picks up randomly a polynomial *g*_*i*_(*x*) = *b*_*i*0_ + *b*_*i*1_ *x* + ⋯+*b*_*it*−1_ *x* ^*t*−1^ ∈ *ℤ*_*q*_[*x*] of degree ≤*t* − 1 such that *g*_*i*_(0) = *b*_*i*0_. Note that the polynomials *f*_*i*_(*x*), *g*_*i*_(*x*), despite having the same degree, are different.
  - It computes and broadcasts *b*_*ij*_ *P*_1_ and sends *g*_*i*_(*j*) to each *E*_*j*_ for *j* = 1,2,…, *n*, *j* ≠ *i*.
7. After *E*_*i*_ receives *g*_*j*_(*i*) from entity *E*_*j*_, *j* = 1,2,…, *n*, *j* ≠ *i*, it does the following:
  - *E*_*i*_ verifies *g*_*j*_(*i*)*P*_1_ by checking that *g*_*j*_(*i*)*P*_1_ = (∑_*k*=0_^*t*−1^ *i* ^*k*^ *b*_*jk*_)*P*_1_. If the check fails, *E*_*i*_ broadcasts a complaint against *E*_*j*_.
  - It computes its private share *s*_*i*_ = ∑_*k*=1_^*n*^ *g*_*k*_(*i*) and keeps it in secret. The element *s*_*i*_ can be regarded as an element of *ℤ*_*q*_.
  - Then, each *E*_*j*_ calculates its public share *Q*_*i*_ = *s*_*i*_ *P*_1_ and computes the public verification key *Q* = (∑_*i*=1_^*n*^ *b*_*i*0_)*P*_1_.
8. With the above calculations, the public key is *Q* = (∑_*i*=1_^*n*^ *b*_*i*0_)*P*_1_; it means that *Q* = *sP*_1_ and its respective private key that is distributed to every entity *E*_*i*_ is *s* = ∑_*i*=1_^*n*^ *b*_*i*0_.

### 3.2. Voting Phase

1. Let $\hat{e}:G_{1}\times G_{1}\to G_{2}$ be the bilinear pairing mentioned above. To encrypt a vote as a message, the voter chooses an option *v* and selects 0 ≠ *r* ∈ *ℤ*_*q*_. Then, it codifies *v* as an element of {0,1}^*n*^. After that, the voter selects any *P*_pub_ID__ and computes one scalar multiplication and one bilinear pairing obtaining the encrypted vote given by (*U*, *W*), where *U* = *rP*_1_ ∈ *G*_1_ and $W=v⊕H_{2}(\hat{e}{\left(P_{pub},P_{{pub}_{ID}}\right)}^{r})\in {\left\{0,1\right\}}^{n}$.
2. The voter gets the blinded encrypted vote *v*′ by choosing randomly 0 ≠ *b* ∈ *ℤ*_*q*_ and calculating *v*′ = *b*(*U* + *H*_1_(*W*)). After that, *v*′ is sent to each entity *E*_*i*_ in order to ask for an *i*-shadow-blind signature to each entity *E*_*i*_, with 1 ≤ *i* ≤ *n*.
3. Each entity *E*_*i*_ computes *σ*_*i*_′ = *s*_*i*_ *v*′ and sends it back to the voter. Since *v*′ ∈ *G*_1_, *σ*_*i*_′ ∈ *G*_1_ as well.
4. The voter calculates the* i-shadow-signature* of each entity *E*_*i*_ by computing *σ*_*i*_ = *b* ^−1^ *σ*_*i*_′ = *s*_*i*_(*U* + *H*_1_(*W*)). Since *σ*_*i*_′ is an element of *G*_1_ so is *σ*_*i*_.
5. Considering a storage device, the vote (*U*, *W*) and the* i-shadow-signatures* are stored as (*U*, *W*‖*σ*_1_‖ ⋯ ‖*σ*_*n*_), where *σ*_*i*_ is computed as in the previous step.

### 3.3. Counting Phase

1. To rebuild and verify the signature of each vote, the independent Combining Entity proceeds as follows:
  - It selects a subset *S*⊆{*σ*_1_, *σ*_2_,…, *σ*_*n*_} of *t* shadow-signatures, that is, |*S* | = *t*, and computes *σ* = ∑_*i*∈*S*_ *L*_*i*_ *σ*_*i*_, where *L*_*i*_ denotes the Lagrange coefficient associated with the polynomial *g*_*i*_(*x*) given by *L*_*j*_ = ∏_*j*∈*S*,*j*≠*i*_(−*j*/(*i* − *j*)) ([17]). Observe that in particular ∑_*i*∈*S*_ *s*_*i*_ *L*_*i*_ = *s*.
  - It verifies the signature by checking that $\hat{e}(\sigma ,P_{1})=\hat{e}(U+H_{1}(W),Q)$.
2. To decrypt the votes, the procedure is as follows:
  - Each entity *E*_*i*_ calculates its decryption share $\hat{e}(U,d_{{ID}_{i}})$ for every vote cast and sends to the Combining Entity, who selects a set $T\subseteq \{\hat{e}(U,d_{{ID}_{1}}),\hat{e}(U,d_{{ID}_{2}}),\ldots ,\hat{e}(U,d_{{ID}_{n}})\}$ of *t* decryption shares and computes $g=\prod_{i\in T}\hat{e}{\left(U,d_{{ID}_{i}}\right)}^{I_{i}}$, where *I*_*i*_ denotes the Lagrange coefficient associated with the polynomial *f*_*i*_(*x*) given by *I*_*j*_ = ∏_*j*∈*S*,*j*≠*i*_(−*j*/(*i* − *j*)) ([17]).
3. Once *g* is determined, the vote is decrypted by computing *v* = *W* ⊕ *H*_2_(*g*).
4. The votes are counted and the tally is published. The voter can check if its vote was counted by comparing its receipt with the announced results.

## 4. Properties Analysis

### 4.1. Privacy

The proposed electronic voting protocol meets the privacy property by using a threshold encryption scheme and its respective signature version, which is probably secure under the Computational Bilinear Diffie-Hellman Problem. With this, only the Combining Entity, jointly with at least *t* entities, is the only one who is able to decrypt votes and verify signatures during the counting stage. The correctness is shown as follows from the signature verification in Section 3.3:

(1) e^σ,P1e^∑i∈SLiσi,P1=∏i∈Se^σi,P1Li=∏i∈Se^b−1σi′,P1Li=∏i∈Se^b−1siv′,P1Li=∏si∈Se^siU+H1W,P1Li=∏i∈Se^U+H1W,P1siLi=e^U+H1W,∑i∈SsiLiP1=e^U+H1W,sP1=e^U+H1W,Q

and from the decryption votes, also in Section 3.3:

(2) g∏i∈Te^U,dIDiIi=e^rP1,∑i∈Tai0IiPpubID=e^rP1,dPpubID=e^P1,PpubIDrd=e^dP1,PpubIDr=e^Ppub,PpubIDr.

Then,

(3) $$\begin{array}{c} W⊕H_{2}\left(\;g\;\right)=W⊕H_{2}\left(\;\hat{e}{\left(\;P_{pub},P_{{pub}_{ID}}\;\right)}^{r}\;\right)=v. \end{array}$$

### 4.2. Robustness

The proposed electronic voting protocol meets robustness property by using bilinear properties in such way that each entity *E*_*i*_ has to prove, in a noninteractive way, the equality of two inverses of the isomorphism $f_{P_{1}}=\hat{e}(P_{1},\cdot )$ induced by the bilinear map $\hat{e}$.

To do this, each entity *E*_*i*_ chooses a random *R* ∈ *G*_1_ and computes $w_{1}=\hat{e}(P_{1},R)\in G_{2}$, $w_{2}=\hat{e}(U,R)\in G_{2}$ and a hash *e* of the tuple $\left(\hat{e}(U,d_{{ID}_{i}}\right)$, $\hat{e}(P_{pub},P_{{pub}_{ID}}),w_{1},w_{2})$.

Then, entity *E*_*i*_ computes *V* = *R* + *e*_*d*ID_*i*__ ∈ *G*_1_ and joins the tuple (*w*_1_, *w*_2_, *e*, *V*) to its share in order that other entities *E*_*i*_ can check that

(4) e^P1,Ve^P1,Re^Ppub,PpubIDe,e^U,V=e^U,Re^U,dIDie.

Both equalities hold as we can see as follows:

(5) e^P1,Ve^P1,Re^Ppubi,PpubIDe,e^P1,R+edIDi=e^P1,Re^Ppubi,PpubIDe,e^P1,Re^P1,dIDie=e^P1,Re^diP1,dIDidi−1e,e^P1,Re^P1,dIDie=e^P1,Re^P1,dIDie,e^U,V=e^U,Re^U,dIDie,e^U,R+edIDi=e^U,Re^U,dIDie,e^U,Re^U,edIDie=e^U,Re^U,dIDie,e^U,Re^U,dIDie=e^U,Re^U,dIDie.

### 4.3. Security Analysis

In the proposed protocol we assume that any attacker who wishes to break the privacy in the proposed electronic voting protocol is fully aware of the public key and any algorithms that may be used as part of the protocol. The information that is denied to the attacker is the private key for encryption during the voting phases.

The nature of the relation between the public and private keys means that it is possible for any asymmetric scheme to achieve a perfect notion of security. Public keys, by definition, must contain enough information to compute their associated private key. In such case it may be theoretically possible to recover the private key from the public key; it is not computationally feasible to do so. Considering that and that we cannot derive definite mathematical statements about the security of the protocol, we do prove that a reduction exists between the difficulty of breaking the protocol and the difficulty of solving a well-studied mathematical problem.

The reductionist approach is used to prove the security in our protocol relying on assumptions about the hardness of some mathematical problems. All of this is made in order to prove its security. We give some definitions as follows.

**Definition 1 .**

Given two groups *G*_1_ and *G*_2_ of the same prime order *q*, a bilinear map $\hat{e}:G_{1}\times G_{1}\to G_{2}$, and a generator *g* of *G*_1_, the Decisional Bilinear Diffie-Hellman Problem (DBDHP) in ${(G}_{1},G_{2},\hat{e})$ is to decide whether $h=\hat{e}{\left(g,g\right)}^{abc}$ given (*g*, *g* ^*a*^, *g* ^*b*^, *g* ^*c*^) ∈ *G*_1_^4^ and an element *h* ∈ *G*_2_.

**Definition 2 .**

Given two groups *G*_1_ and *G*_2_ of the same prime order *q*, a bilinear map $\hat{e}:G_{1}\times G_{1}\to G_{2}$, and a generator *g* of *G*_1_, the Computational Bilinear Diffie-Hellman Problem (CBDHP) in ${(G}_{1},G_{2},\hat{e})$ is to compute $h=\hat{e}{\left(g,g\right)}^{abc}$ given (*g*, *g* ^*a*^, *g* ^*b*^, *g* ^*c*^) ∈ *G*_1_^4^.

In other words, security of proposed protocol is based on hardness assumptions for problems in groups equipped with a pairing. The advantage of solving such assumptions is given as follows.

**Definition 3 .**

The advantage of an algorithm *A* in solving the Bilinear Diffie-Hellman Problem (BDHP) in (*G*_1_, *G*_2_) is

(6) $$\begin{array}{c} {Adv}_{A}^{BDHP}\left(\;k\;\right)=\mathrm{Pr}\left[\;A\left(\;g,g^{a},g^{b},g^{c}\;\right)=\hat{e}{\left(\;g,g\;\right)}^{abc}\;\right], \end{array}$$

where $a,b,c\overset{\text{\$}}{\leftarrow }Z_{q}^{*}$ and we assume that parameters (*G*_1_, *G*_2_, *e*, *q*, *g*) as output by the algorithm* PairingGen* on input 1^*k*^ are given to *A* as additional inputs. We say that the BDHP is hard in (*G*_1_, *G*_2_) if no Polynomial Time Algorithm that solves the BDHP in (*G*_1_, *G*_2_) has a nonnegligible advantage, as a function of the security parameter *k*.

**Definition 4 .**

The advantage of an algorithm *A* in solving the Decisional Bilinear Diffie-Hellman Problem (DBDHP) in (*G*_1_, *G*_2_) is

(7) AdvADBDHPk=Pr⁡Ag,ga,gb,gc,e^g,gabc=1−Pr⁡Ag,ga,gb,gc,Z=1,

where $a,b,c\overset{\text{\$}}{\leftarrow }Z_{q}^{*}$ and $Z\overset{\text{\$}}{\leftarrow }G_{2}$. Moreover, we assume that parameters (*G*_1_, *G*_2_, *e*, *q*, *g*) as output by the algorithm* PairingGen* on input 1^*k*^ are given to *A* as additional inputs. We say that the DBDHP is hard in (*G*_1_, *G*_2_) if no Polynomial Time Algorithm that solves the DBDHP in (*G*_1_, *G*_2_) has a nonnegligible advantage, as a function of the security parameter *k*.

**Definition 5 .**

The advantage of an algorithm *A* in solving the Computational Bilinear Diffie-Hellman Problem (CBDHP) in (*G*_1_, *G*_2_) is

(8) AdvACBDHPk=Pr⁡Ag,ga,gb,gc,e^g,gabc=h,

where $a,b,c\overset{\text{\$}}{\leftarrow }Z_{q}^{*}$ and $h=\hat{e}{\left(g,g\right)}^{abc}\overset{\text{\$}}{\leftarrow }G_{2}$. Moreover, we assume that parameters (*G*_1_, *G*_2_, *e*, *q*, *g*) as output by the algorithm* PairingGen* on input 1^*k*^ are given to *A* as additional inputs. We say that the CBDHP is hard in (*G*_1_, *G*_2_) if no Polynomial Time Algorithm that solves the CBDHP in (*G*_1_, *G*_2_) has a nonnegligible advantage, as a function of the security parameter *k*.

Considering the aforementioned, to break our protocol from the privacy point of view, first of all, attacker must break the atomic primitives our cryptographic protocol is based on in addition to getting nonnegligible advantage in the above definitions.

## 5. Results

In order to get a comparison between the proposed protocol and related work, results are shown from two points of view; Table 1 shows the first one, which is viewed from the total number of PKI components that the proposed protocol would use to develop a voting process. In such table PKI Component 1 and PKI Component 2 mean certification and trust authorities, respectively. Both of them are main components in a PKI. In that table it is possible to see that the number of components required increases depending on the number of voters participating in the voting protocol. Moreover, the proposed electronic voting protocol meets privacy and robustness based on Diffie-Hellman problems, which become as secure as [5] and more secure than [1–4], as [5] reports. In this sense CBDHP means Computational Bilinear Diffie-Hellman Problem.

On the other hand, the second point of view is from the computations needed to develop the proposed protocol, which depends on the number of cryptographic operations used in comparison with the proposed one. Operations considered are modular addition (+), modular multiplication (*∗*), exponentiation (*x* ^*y*^), inversion (*x* ^−1^), point addition (*P* + *Q*), and scalar multiplication (*nP*). Moreover, *v* means voter and parameter *n* represents the total number of shareholders who participate during the voting process with 1 ≤ *i* ≤ *n* and *t* denotes the threshold that the voting protocol considers for counting stage. It is important to say that our protocol involves operations based on groups, finite fields, and field extensions, which are made by using polynomials to represent the field elements that bilinear maps use.

In Table 2 it is possible to see that even though the proposed protocol does not involve exponentiations and point additions, it does use several computations of bilinear maps, which involves additions and multiplications over a finite field and its extensions, a technique called tower fields. However, even though the proposed protocol has the highest computational cost, bilinear maps can be addressed by using cryptoaccelerators, which efficiently develops such kind of cryptographic operations. The inclusion of such processors is considered to be cheaper and preferred than the components of a Public Key Infrastructure.

## 6. Conclusion

Electronic voting protocols that include as main construction blocks blind signatures and homomorphic and secret sharing techniques have been developed in last years. In this paper we present a protocol that is based on blind signatures and secret sharing techniques, using blind signatures and encryption schemes as the main construction blocks. The main difference with protocols proposed to date is that its functionality is based on bilinear maps and secret sharing schemes, which are used jointly with their respective properties to meet expectations of privacy and robustness. Bilinear maps develop high cost operations which can be addressed by using cryptoaccelerators to efficiently develop this sort of operations. As a result, we eliminate the need of implementing a Public Key Infrastructure (PKI).

In addition the proposed protocol is based on the difficulty of solving the Computational Diffie-Hellman Problem (CDHP) and the Bilinear Diffie-Hellman Problem (BDHP); due to its construction it can be found on supersingular elliptic curves or hyperelliptic curves over finite fields; as a consequence no algorithm exists as yet capable of solving such problems in polynomial time.

According to what was mentioned above, it is easy to see that proposed protocol highlights the balance between security and efficiency. In other words, from the security point of view, the proposed protocol is based on the difficulty of solving the Computational Diffie-Hellman Problem (CDHP) and the Bilinear Diffie-Hellman Problem (BDHP). From the efficiency point of view, we eliminate the need of implementing the components of a Public Key Infrastructure (PKI) and leave as consideration the development of cryptographic operations by using cryptoaccelerators.

The protocol presented here could be used, for instance, in a voting system based on Direct Recording Electronic (DRE) systems, which provides authentication of the voter's identity based on official documents presented to the electoral authority.

Moreover, the voter's receipt could be used to meet requirements of verifiability and accuracy. Thus, in order to verify if the votes were recorded and counted, the receipt should appear on a bulletin board in which it is displayed together with the final tally. If any voter does not find his/her hash value on the bulletin board, he/she can register a complaint with election officials.

## Figures and Tables

**Table 1:** Proposed protocol does not need any component of a PKI.

Protocol	PKI Component 1	PKI Component 2	Privacy and robustness based on
Cramer et al. [1]	0	0	DHP
Mu et al. [2]	1*∗V*	0	DHP
Ohkubo et al. [3]	1*∗v*	0	DHP
Baudron et al. [4]	1*∗L*	0	RC
Gallegos-García et al. [5]	0	1	CBDHP
Proposed protocol	0	0	CBDHP

**Table 2:** Cryptographic operations developed in the proposed protocol.

Op.	Ohkubo et al. [3]	Cramer et al. [1]	Baudron et al. [4]	Mu et al. [2]	Gallegos-García et al. [5]	Proposed protocol
+	3	1	2(*i* − 2) + 2	1	0	(*t* − 1)*∗*2
*x*	16 + (*t* − 1)	12 + (*i* − 1)	*L∗*10 + 8 + 2(*t* − 1)	6	0	(*t* − 1)*∗*2
*x* ^*y*^	13	19 + *n* + *n∗i*	*L*(*n*!+13) + 9 + *t*	11	0	0
*x* ^−1^	2	3	*L∗*2	1	1	1
*P* + *Q*	NA	NA	NA	NA	0	0
*nP*	NA	NA	NA	NA	2 + 1*∗v* + 3*∗i*	2*∗*((4*∗i*)+(1*∗j*)+(1*∗j∗t* − 1)+(1*∗i∗n*))+(1*∗i*)+(2*∗v*)*∗*(1*∗v∗i*)
Hash	5	NA	NA	NA	(1*∗i*)+(3*∗v*)	(1*∗i*)+(3*∗v*)
$\hat{e}$	0	0	0	0	(1*∗v*)+(1*∗i*) + 2	(1*∗v*)+(1*∗i*) + 2
